# 3-Arylaziridine-2-carboxylic Acid Derivatives and (3-Arylaziridin-2-yl)ketones: The Aziridination Approaches

**DOI:** 10.3390/ijms22189861

**Published:** 2021-09-13

**Authors:** Boriss Strumfs, Romans Uljanovs, Kirils Velikijs, Peteris Trapencieris, Ilze Strumfa

**Affiliations:** 1 Latvian Institute of Organic Synthesis, 21 Aizkraukles Street, LV-1006 Riga, Latvia; boriss@osi.lv (B.S.); peteris@osi.lv (P.T.); 2 Department of Pathology, Riga Stradins University, 16 Dzirciema Street, LV-1007 Riga, Latvia; Romans.Uljanovs@rsu.lv (R.U.); Kirils.Velikijs@rsu.lv (K.V.)

**Keywords:** aziridines, aziridination, imines, carbenes, nitrenes

## Abstract

Aziridination reactions represent a powerful tool in aziridine synthesis. Significant progress has been achieved in this field in the last decades, whereas highly functionalized aziridines including 3-arylated aziridine-2-carbonyl compounds play an important role in both medical and synthetic chemistry. For the reasons listed, in the current review we have focused on the ways to obtain 3-arylated aziridines and on the recent advances (mainly since the year 2000) in the methodology of the synthesis of these compounds via aziridination.

## 1. Introduction

The finding of new potential anti-cancer and antiviral drugs, as well as the development of efficient methods for synthesizing them and their appropriate building blocks, is one of the most important problems in medical chemistry. Due to the electrophilic nature of the aziridine ring, derivatives of aziridine-2-carboxylic acid react with various nucleophiles, therefore becoming interesting synthetic substrates in order to create different amino acids, alkanolamines, and heterocyclic compounds [1]. Some derivatives of aziridine-2-carboxylic acid, namely, imexon, azimexon [2], and leakadine [3] were developed as anti-tumour agents and have shown anti-cancer immunomodulatory activity. 

**Scheme 1:**
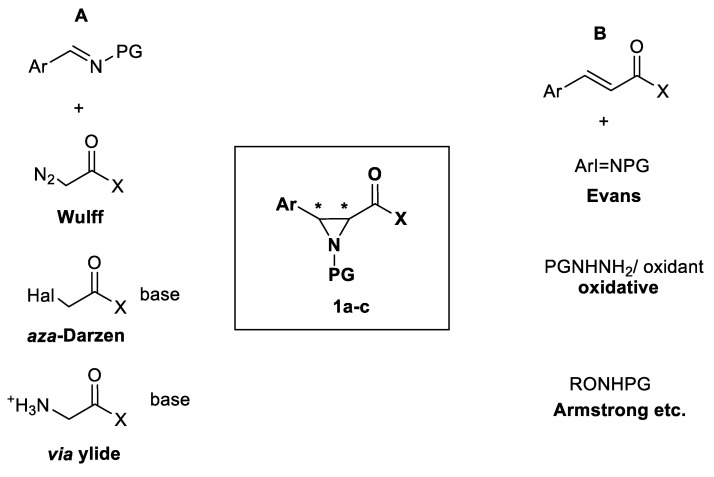
The general pathways of aziridination. (**A**) carbon insertion in the imine double bond via carbene sources; (**B**) ni-trogen insertion in the olefin double bond via nitrene sources

It is also known that derivatives of aromatic α,β-unsaturated carboxylic acids, such as caffeic acid [4] and its esters [5] have demonstrated cytotoxic effects and promotion of apoptosis in lung carcinoma cells. Its analogue, *p*-coumaric acid, has shown an anti-angiogenic effect [6], which is important to stop tumor development. In this light, 3-arylated derivatives of aziridine-2-carboxylic acid **1a**,**b** (Scheme 1) and similar compounds containing an aziridine ring in phenylpropionic acid side chain could be predicted to have anti-cancer properties.

Authors of previous reviews have summarized the recent advances in the synthesis of aziridine-2-carbonyl compounds (Zalubovskis and Ivanova [7]) and in the overall aziridine chemistry and synthesis (Singh [8] and Luisi [9]). The generally known methods to obtain these compounds include aziridination, Gabriel-Cromwell cyclization of aminoalcohols, Diels-Alder cycloaddition to azirines, Baldwin rearrangement, and others. This review is focused on synthesizing 3-aryl substituted aziridines **1a**–**c** (Scheme 1) via aziridination since aziridination has a wide perspective, especially in stereoselective synthesis; it is tolerant towards various functional groups and can be realized in mild conditions which are important for construction of more complex molecules. The structures **1a**–**c** (Scheme 1) have two asymmetric carbons in 2(α-) and 3(β-) positions of aziridine ring, and therefore stereoselectivity of synthetic methods is important. Aziridination has two variations: carbon addition to the imine double bond via carbene sources (Scheme 1A) and nitrogen addition to the olefin double bond via nitrene sources (Scheme 1B).

## 2. Aziridination of Imines (Path A)

This aziridination approach includes:

- Aziridination of imines with a diazo carbene source (AZ reaction);
- Variations of *aza*-Darzen reaction;
- Aziridination with ylides.

### 2.1. Aziridination of Imines with a Diazo Carbene Source (Wulff’s AZ Reaction)

Catalytic asymmetric aziridination (AZ reaction) of imines using a carbene source represents the best explored and most popular group of synthetic methods of 3-arylated aziridines **1a**–**c** (Scheme 1). The most common carbene sources are diazo compounds, e.g., ethyl diazoacetate (EDA) and its analogues.

**Scheme 2:**
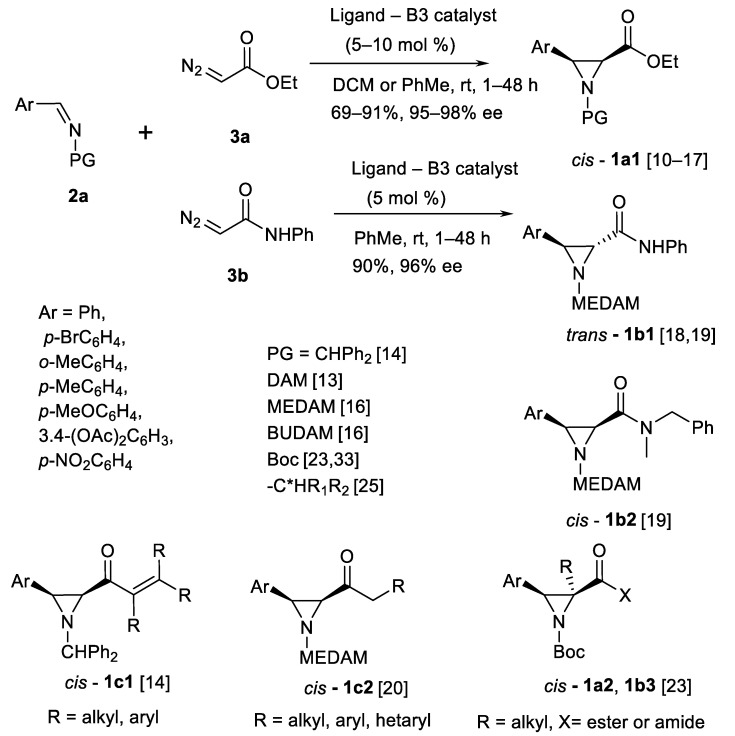
Wulff’s type aziridination (AZ reaction) [10,11,12,13,14,15,16,17,18,19,20,21,22,23,24,25,26,27,28,29,31,32].

**Scheme 3:**
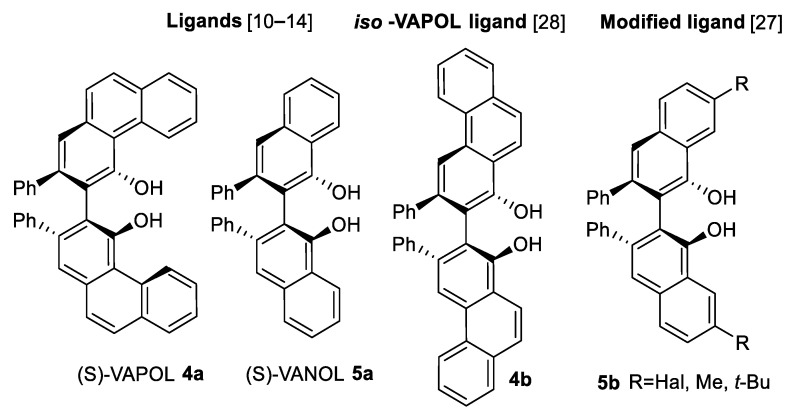
Aryl ligands for Wulff’s aziridination [10,11,12,13,14,27,28].

Fundamental series of research in catalytic C=N bound (imine) aziridination with diazo carbene sources (Scheme 2) was made by Wulff’s and Thurston’s teams [10,11,12,13,14,15,16,17,18,19,20,21,22,23,24,25,26,27,28,29,30,31,32]. A particularly large number of scientific publications have been prepared by the Wulff’s group [10,11,12,13,14,15,16,17,18,19,20,21,22,23,24,25,26,27,28,29,31,32]. At first, it was established that the best catalysts for aryl imine **2a** (Scheme 2) and ethyl diazoacetate (EDA) **3a** (Scheme 2) reactions were complexes of (*S*)-VAPOL **4a** (Scheme 3) and (*S*)-VANOL **5a** ligands (Scheme 3) with B(OPh_3_). *cis*-Aziridine esters **1a1** (Scheme 2) were obtained in good (69–91%) yields and high enantiomeric (ee) purity [10,11,12]. AZ reaction-based synthesis of L-DOPA [10] and (-)-chloroamphenicol [11] and florfenicol [12] have been demonstrated. In the case of dianisylmethyl (DAM) substituent 3-aryl-NH, aziridines were successfully obtained after hydrogenation in 83–100% yields [13]. Instead of ester diazo carbene source **3a**, diazoamide **3b** (Scheme 2) produced *trans*-aziridines **1b1** as illustrated in the Scheme 2 [18].

Aziridinyl vinyl ketones **1c1** (Scheme 2) also have been obtained from the corresponding diazo ketones using AZ reaction in presence of (*S*)-VANOL and (*S*)-VAPOL-derived boron complex catalysts in 19 examples at 35–90% yields [14]. Series of 3–arylated aziridinyl ketones **1c2** (Scheme 2; seven examples, 62–90% yields, 85–99% ee) have been synthesized in tandem acylation-aziridination of trimethylsilyldiazomethane [20] using (*S*)-VAPOL and (*R*)-VANOL ligands. N-Boc-imines **2a** (Scheme 2) are more reactive toward diazo compounds and allow to reach trisubstituted aziridines *cis*-**1a2**, **1b3** shown in the Scheme 2 [23].

**Scheme 4:**
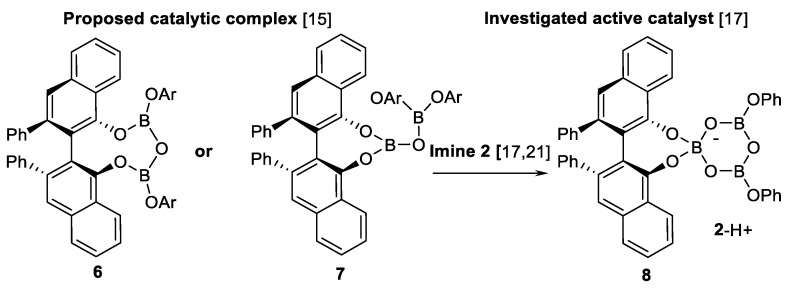
Boroxinate catalyst [15,17,21].

More detailed studies of this AZ reaction were carried out in 2008 [15,16]. The structure of active catalyst was established [15]. This is a pyroborate **6** or **7** (Scheme 4). Reaction with a catalyst **6** and **7** (Scheme 4) has been explored in case of EDA **3a** (Scheme 2) and 12 different imines **2a** (Scheme 2), ee of the obtained 3-aryl aziridines **1a** (Scheme 2) were in the range of 90–95%.

In the subsequent study [16], the active site of the aziridination catalyst “chemzyme” was explored using different N-substituents in the imine **2a** (Scheme 2), and authors found that 3.5-dimethyldianisilmethyl (MEDAM) and 3.5-di-*tert*-butyldianisilmethyl (BUDAM) groups resulted in the best asymmetric inductions in AZ reaction. Further, evidence of cyclic self-assembled boroxinate Brønsted acid ion pair **8** (Scheme 4) acting as an active catalyst in the asymmetric AZ reaction has been reported [17].

Control of diastereo- and enantioselection of chiral VAPOL/VANOL-based polyborate Brønsted acid catalyst-based AZ reactions has been studied [18,19]. In case of diazoamide **3b**, *trans* aziridines (Scheme 2; **1b**) can be obtained in reasonably high yields (90%) and enantioselectivity reaching 96% [18]. MEDAM group is the best imine **2a** (Scheme 2) N-substituent in this reaction. Also, *cis* aziridine amides **1b** (Scheme 2) are obtained if the amide group is more sterically hindered [19]. Product stereochemistry-determining transition states have been studied both experimentally and in silico to obtain universal methodology of catalytic AZ reaction [19].

The previously described results have been summarized by Mukherjee et al. [21]. The N-substituents have been compared, and it was found that MEDAM imines **2** (Scheme 2) showed 96–97% ee in resulting aziridines **1a**,**b** (Scheme 2). Imines **2a** (Scheme 2) derived from seven different aryl aldehydes were examined in aziridine ester **1a** and amide **1b** (Scheme 2) synthesis.

Structure of the self-assembled boroxinate-imine complex **8** (Scheme 4) has been characterized by X-ray diffraction in case of two “chemzyme”-substrate complexes [22]. A practical gram-scale methodology of boroxinate Brønsted acid-catalyzed AZ reaction has been developed [24]. The further steps in developing this asymmetric aziridination tool were experiments with double stereodifferentiation using imines **2** (Scheme 2) obtained from chiral amines (chiral PG) [25] and one-pot five-component reaction protocol: Base-induced formation of boroxinate catalyst **8** (Scheme 4) followed with subsequent addition of diazo compound **3** was replaced with simultaneous addition of all reagents [26].

The further increase of AZ reaction enantioselectivity was realized through insertion of substituents in 7, 7′ positions of biaryl ligand **5b** as depicted in the Scheme 3 [27]. Improving of catalyst ligand included developing of *iso*-VAPOL ligand **4b** illustrated in the Scheme 3 [28]. This ligand was an isomer of VAPOL **4a** (Scheme 3) but had a chiral pocket of VANOL **5a** (Scheme 3) and was available from much cheaper starting materials.

**Scheme 5:**
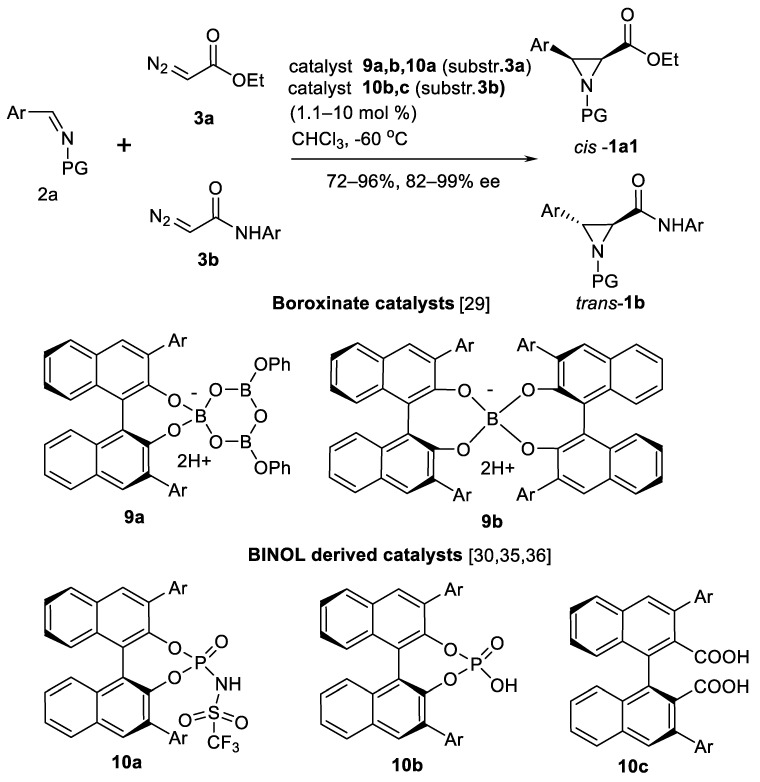
Boroxinate and BINOL catalysts in AZ reaction [29,30,31,32,33,34,35,36].

In 2017, Wulff’s group continued to study AZ reaction using closely related BINOL catalysts **9a** and **9b** represented in the Scheme 5 [29]. Different borate ester structures were investigated. Boroxinate **9a** and spiro borate **9b** (Scheme 5) yielded opposite asymmetric inductions in aziridine *cis*-**1a1** (Scheme 2) formation.

A series (12 examples) of esters *cis*-**1a1** (Scheme 2) were obtained by Thurston’s group using BINOL-derived Brønsted acid catalyst **10a** (Scheme 5) [30]. Multi-component variation of AZ reaction was carried out employing aromatic and heteroaromatic aldehydes. Other examples of multi-component AZ reaction approach involving the above mentioned VAPOL and BOROX catalysts are also known [31,32]. Optimal reaction protocol for aromatic aldehydes in synthesis of aziridines *trans*-**1b** (Scheme 2) was found [32].

**Scheme 6:**
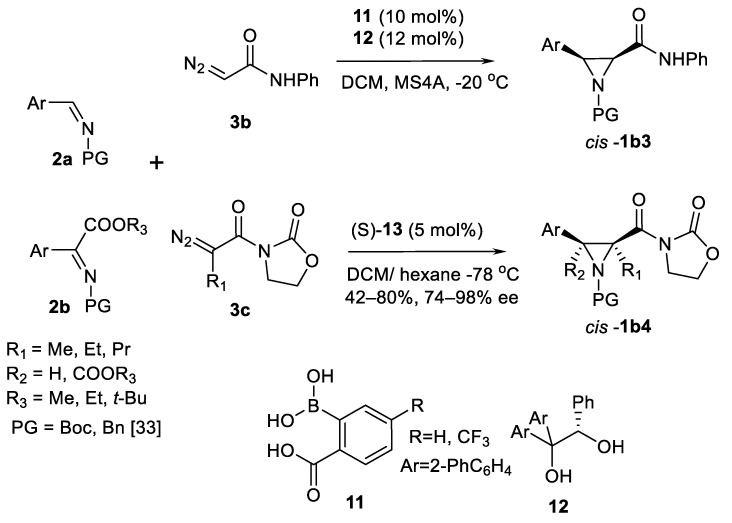
AZ reaction with Maruoka’s catalysts [33].

Another boron Brønsted acid catalyst system—arylboronic acid **11** and chiral diol ligand **12** in situ assembled boronate ester (Scheme 6) was developed by Maruoka and co-workers [33]. Aziridine amides *cis*-**1b3** (Scheme 6) were obtained in good yields and high enantioselectivities (22 examples). In case of α-diazoacyl oxazolidinones **3c** (Scheme 6) as carbene sources and chiral N-triflyl phosphoramide Brønsted acid catalyst **10a** (Ar = Ph) [34] highly substituted oxazolidinone aziridine amides *cis*-**1b4** (Scheme 6) were obtained in good (77–91%) chemical yields and usually >80% ee. Series of different 3–aryl substituents were demonstrated to be useful in these reactions in 24 examples. Reasonable *trans*-selectivity was reached in this type of reactions with chiral phosphoric acids **10b** (Ar = 9-Anthryl) [35] and **10c** (Ar = 2.4.6-Me_3_-C_6_H_2_; Scheme 5) [36] as a catalyst to obtain amides *trans*-**1b3** in excellent yields (89–97%) and enantioselectivity (88–98%), shown in 16 examples [35] and 14 examples [36], respectively.

**Scheme 7:**
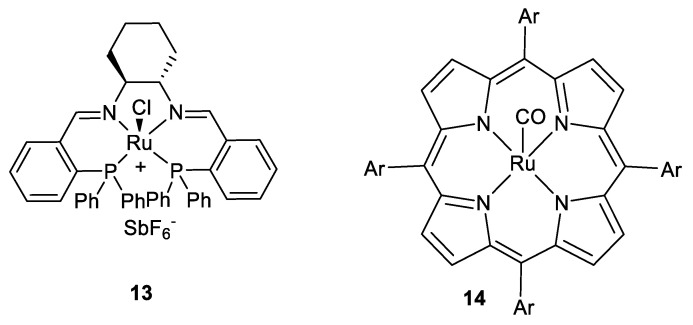
Ruthenium catalysts for the AZ reaction [37,38,39].

Enantioselective AZ reaction using another type of catalyst, namely, a ruthenium complex **13** (Scheme 7), was investigated by Mezzeti and co-authors [37,38].

Six examples of β-aryl esters *cis*-**1a** (PG=CHPh_2_) were demonstrated in moderate yields (9–33%) and good ee (75–93%). Another ruthenium complex (Scheme 7; **14**) as a catalyst has been used in the same AZ reaction, leading to yields of esters *cis*-**1a** (16 examples) up to 86% [39] but the stereoselectivity was moderate, typically 2.5:1. Iron-pyBOX complexes [40] and rhodium-benbox complexes (ten examples [41]) as AZ reaction catalysts at room temperature were reported earlier, but results (yields, ee) were moderately good.

**Scheme 8:**
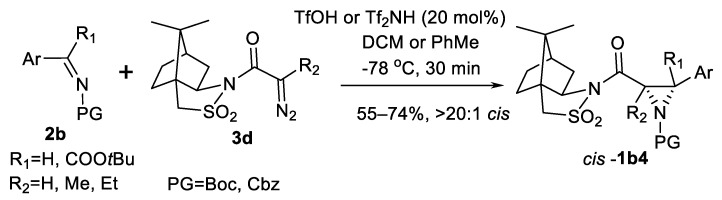
Aziridination with a chiral diazonium reagent [42,43].

The use of a chiral diazonium compound (Scheme 8) represents an interesting asymmetric variation of AZ reaction [42,43]. Thus, N-α-diazoacyl camphorsultams **3d** demonstrate good results in reaction with imines **2b** to obtain β-mono- and disubstituted aziridine-2-carboxamides *cis*-**1b4**.

**Scheme 9:**
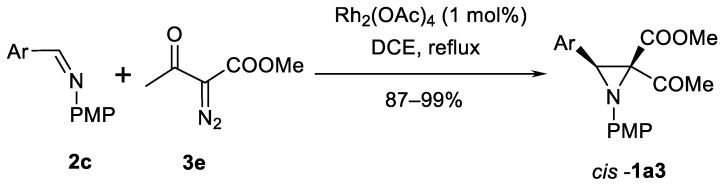
Synthesis of 3-arylaziridine ketoesters [44].

Diazoacetates **3e** are useful substrates for synthesis of highly functionalized β-aryl aziridine ketoesters *cis*-**1a3** as shown in the Scheme 9 [44]. Six successful examples with different arylimines were presented. Unfortunately, if Ar = *p*-NO_2_Ph, or 2-pyridyl, no reaction was observed. On the other hand, some examples of products *cis*-**1a3** with another ester groups and 2.2-diesters instead of ketoester were demonstrated.

Ionic liquids have been successfully tested for *cis*-selective AZ reactions [45,46]. Reactions of aromatic aldimines were carried out in bmim PF_6_ at room temperature [45] and in the same liquids in multi-component variation with 2 mol% of Bi(OTf)_3_ or 5 mol% of Sc(OTf)_3_ catalyst [46] addition. The yields of aziridines *cis*-**1a** in both studies exceeded 80%. Ten [45] and twelve [46] examples were demonstrated, respectively.

**Scheme 10:**
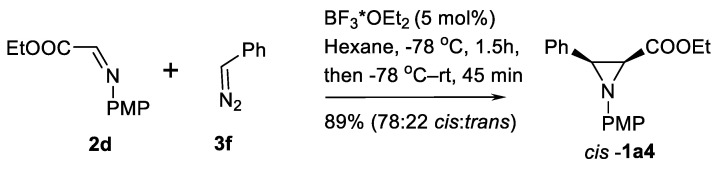
Lewis acid-catalyzed aziridination [47].

Other simple catalysts for imine C=N bound aziridination with diazo compounds include BF_3_*OEt_2_ [47,48], montmorillonite K-10 [49], LiClO_4_ [50] and Rh_2_(OAc)_4_ [44,51]. The Lewis acid BF_3_*OEt_2_-catalyzed reaction of imino ester **2d** and phenyldiazomethane **3f** produces the aziridine ester *cis*-**1a4** in good yield [47] without enamino- and dimeric byproducts (Scheme 10).

Sugar-derived imine also was successfully aziridinated under BF_3_*OEt_2_ catalysis [48]. The montmorillonite K-10 catalysis in imine-EDA aziridine-forming reaction is characterised by high *cis*-selectivity and good yields (15 examples). The demonstrated procedure is very simple, namely, reactions were performed at room temperature with EDA as solvent [49]. A similar method with LiClO_4_ catalysis allows to obtain broad spectrum (18 examples) of 3-aryl aziridine esters *cis*-**1a** at room temperature in acetonitrile over 4.5–7.5 h at >75% chemical yields and good stereoselectivity [50].

Organocatalytic variations of AZ reaction are also known. In presence of pyridinium salts as Brønsted acid organocatalysts *cis*-selective synthesis of esters *cis*-**1a** was demonstrated [52,53]. The best results show 10 mol% of pyridinium triflate [53]. Synthesis of 21 examples of different 3-aryl aziridine-2-carboxylates **1a** bearing substituted phenyls and 2-pyridils in β-position has been demonstrated (70–99% yields). Polymer-supported pyridinium salt also was reported as an effective catalyst (80% yield of product), and three-component one-pot process was performed.

**Scheme 11:**
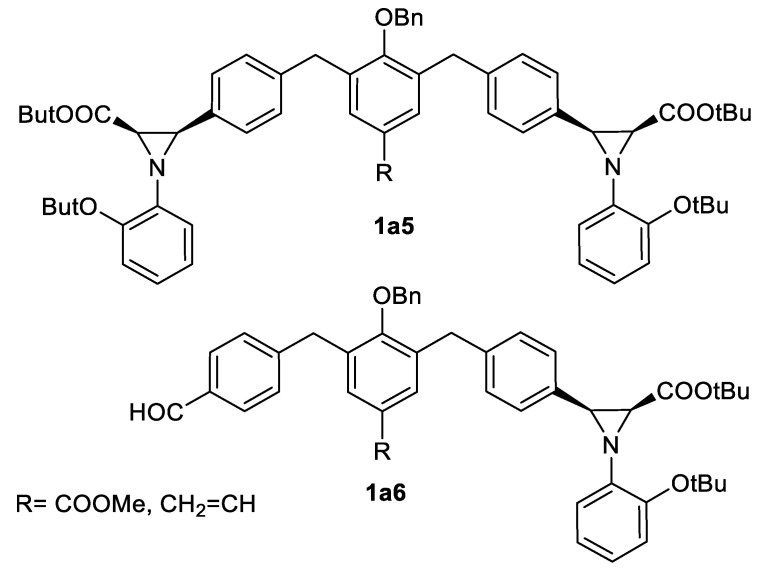
Pyridinium triflate-catalyzed aziridination products [56].

In addition, N-fluoropyridinium triflate (fluoronium ion catalyst) has been described as an organocatalyst in eight examples of these reactions [54], and the reached results were comparable with the previously listed examples. A source of cation radical, tris-(4-bromophenyl) aminium hexacloroantimonate (TBPA^+^ SbCl_6_^-^), was an effective initiator of EDA **3a** addition to aryl imines **2** in 13 examples [55]. *tert*-Butyl diazoacetate addition to one-pot-generated aldimines **2** under pyridinium triflate catalysis was successfully employed to obtain more complex 3-aryl aziridine structures **1a5**, **1a6** shown in the Scheme 11 [56]. 

An interesting variation of imine aziridination was EDA-aziridination of in situ generated iminium ion [57], generated from α-aminonitriles.

### 2.2. Aziridination of Imines with Other Carbene Precursors

Other carbene precursors or carbene-like species can be added to imines. These species include:

- Active methylene compounds;
- Enolates derived from α-bromoesters and bases;
- Lithiated enamines;
- Guanidinium, ammonium or sulfonium ylides.

#### 2.2.1. Variations of *aza*-Darzen Reaction 

The most frequently used method is the addition of enolates derived from α-bromoesters to various imines (*aza*-Darzen reaction variations).

**Scheme 12:**
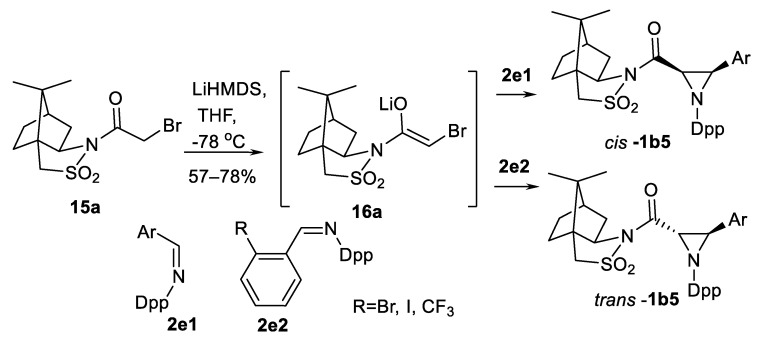
Sweeney’s *aza*-Darzen type aziridination [58,59].

The sources of chirality are chiral N-substituents in imines or enolates. Use of chiral enolates and N-diphenylphosphinylimines were explored by Sweeney and co-authors [58,59]. Thus, enolate **16a**, generated from 2*R*-N-bromoacethylcamphorsultam **15a** (Scheme 12) reacts with N-diphenylphosphinyl aldimines **2e1** leading to 3-arylaziridine-2-carboxamides *cis*-**1b5**.

Chemical yields are good (57–78%) and stereoselectivity excellent, >95% dr in 14 demonstrated examples [58]. However, in some cases depending of the imine aryl substituent structure (using imines **2e2**) inversion of stereochemistry has been observed and aziridines *trans*-**1b5** obtained [59]. The mechanism of reaction and transition states were elucidated.

Another approach to chiral 3-aryl aziridine-2-carboxylic acid derivatives by asymmetric *aza*-Darzen type reaction is the use of chiral moiety in the imine component.

**Scheme 13:**
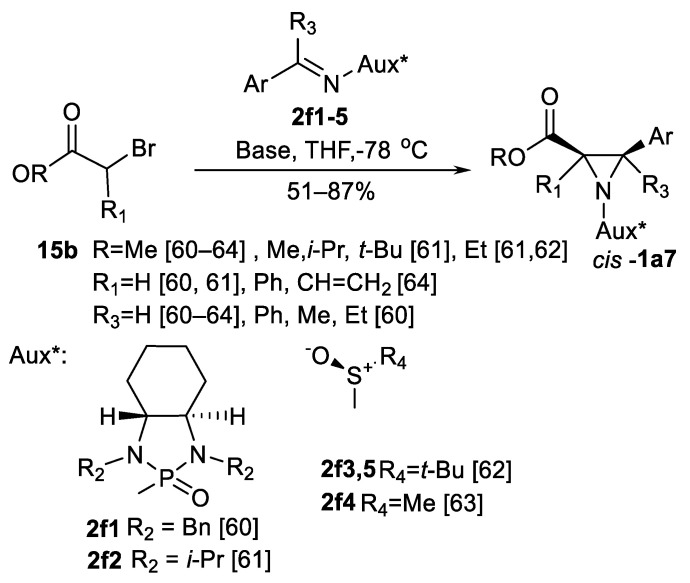
*aza*-Darzen aziridinations with chiral imines [60,61,62,63,64].

There are chiral N-phosphinyl imines **2f1**, **2f2** [60,61] and chiral sulfimines **2f3**, **2f4** [62,63,64] presented (Scheme 13). N-phosphinyl imines **2f1** have been successfully used in *aza*-Darzen reaction with enolates generated from esters **15b** in ten examples of aziridines *cis*-**1a7** in 72–82% chemical yields and >80% de [60]. Better results have been achieved by imines **2f2**: 17 examples of aziridines *cis*-**1a7** with various 3–aryl substituents and four examples of different esters **1a7** (Ar = Ph) have been obtained in good chemical yields (51–87%) and >98% *cis*- selectivity [61]. Chiral *tert*-butanesulfinyl aldimines **2f3**, **2f4** and ketimines **2f5** were used in series of aziridines *cis*-**1a7** synthesis [62]. Corresponding 3-aryl and 3.3-diaryl products were obtained in moderately good *cis*-selectivity (71:29–98:2); eight examples of 3-aryl products *cis*-**1a7** were demonstrated. Isolated examples of S-mesitylsulfinyl imines **2f4** have been employed in aziridine *cis*-**1a7** synthesis [63]. Trisubstituted 3-aryl aziridine-2-carboxylates *cis*-**1a7** were obtained from substituted 2-bromoesters **15b** and *tert*-butane sulfinyl aldimines **2f3** [64] in >60% chemical yields and >98% de (five examples).

**Scheme 14:**
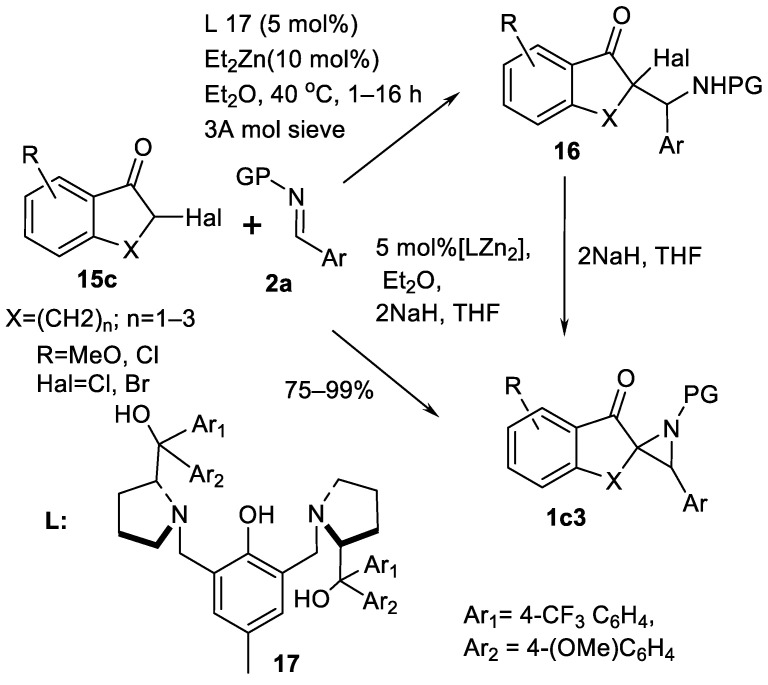
Spirocyclic aziridine products via *aza*-Darzen aziridination [65].

Modified *aza*-Darzen type protocol allows to obtain trisubstituted spirocyclic 3-aryl aziridinyl ketones **1c3** (Scheme 14) from cyclic halogenated ketones **15c** illustrated in the Scheme 14 [65]. Two protocols—one-step direct approach to ketones **1c3** (Scheme 14) and two-step synthesis through an addition product, namely, tertiary halogenide **16** (Scheme 14)—were realized. Chloro- and bromoketones **15c** (Scheme 14) were used, and the catalyst was Zn-ligand **17** (Scheme 14) complex. Reactions can be run in gram-scale with small amounts of catalyst. Chemical yields of products (30 examples) are 75–99% in >20:1 dr.

**Scheme 15:**
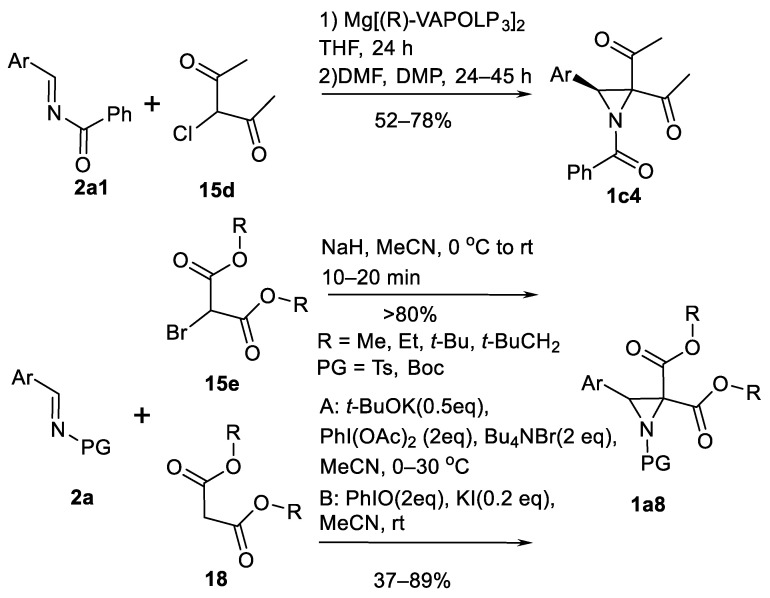
Diketones and diesters in *aza*-Darzen aziridination [66,67,68,69].

In a similar way, α-chloro-1.3-diketones **1c4** (Scheme 15) were used as enolate precursors in *aza*-Darzen type synthesis [66] in reactions with N-benzoylarylaldimines **2a1** under (*R*)-VAPOL magnesium phosphonate salt catalysis (Scheme 15). In case of electron deficient imines **2a** (Scheme 15), no strong bases are necessary and aziridines **1c4** (Scheme 15) form in 52–78% yield and 57–92% ee.

3-Arylated aziridine diesters **1a8** (Scheme 15) also can be prepared in *aza*-Darzen reaction [67]. Racemic aziridines **1a8** (Scheme 15) are obtained in >80% yields from imines **2a** (Scheme 15) and bromomalonates **15e** (Scheme 15). Useful N-substituent for these reactions was tosyl (24 examples) but N-Boc aziridine diester **1a8** (Scheme 15) also can be prepared from N-Boc imine **2a** (Scheme 15). N-tosyl imines **2a** (Scheme 15) can react with activated methylene compounds **18** (Scheme 15) in iodine (III) induced aziridinations to form aziridines **1a8** as shown in the Scheme 15 [68,69]. Iodobenzene diacetate—tetrabutylammonium bromide system was reported first [68]. Aziridines **1a8** (Scheme 15) can form from malonates **18** (Scheme 15), acetyl-, cyano- and nitroacetic acid esters to obtain symmetrical and unsymmetrical products **1a8** (Scheme 15) in 37–89% yields. The further study showed that PhIO-KI system is a better oxidative iodine (III) additive for these reactions [69]. In this case, aziridines **1a8** (Scheme 15) are obtained in >75% yields.

**Scheme 16:**
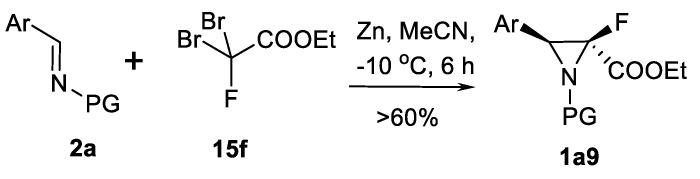
Reformatsky type *aza*-Darzen aziridination [70].

Reformatsky type *aza*-Darzen reaction variation using imines **2a**, inactivated zinc metal and fluorodibromoacetate **15f** is successfully used for access to 3-arylated 2-fluoro aziridine-2-carboxylates **1a9** as shown in the Scheme 16 [70].

Yields up to 60% (determined by ^19^FNMR) and up to 80% *syn* products were obtained in ten examples.

**Scheme 17:**
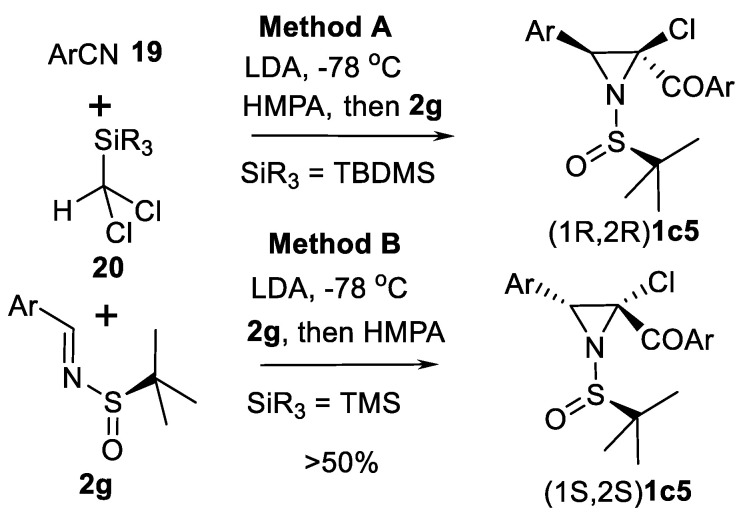
Cascade aziridination examples [71].

More complicated cascade reactions (Scheme 17) which allowed stereoselective obtaining of 3-arylated 2-chloro aziridines was demonstrated by Xu and co-authors [71]. This cascade coupling included nucleophilic addition of anion generated from silyldichloromethane **20** and nitriles **19** in presence of LDA and subsequent [1,3] -*aza*-Brooke rearrangement to give α-N-silyl imines in equilibrium with 1-azaenolate equivalents. These species were then trapped by imines **2g** in an *aza*-Darzen type reaction to give aziridines **1c5** in good (up to 50%) yields and up to 10:1 selectivity demonstrated in 19 cases for each method. Remarkably, stereoselectivity strictly depends on the silyl group and the order of addition of **2g** and HMPA (method A or B).

#### 2.2.2. Ylides as Carbon Sources

**Scheme 18:**
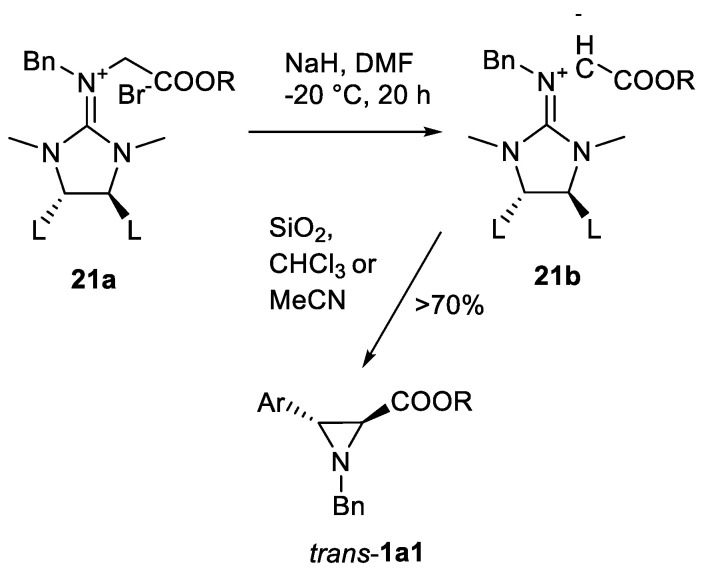
Ishikava’s aziridination [72,73,74,75,76,77].

An interesting variation of C=N double-bond aziridination are aziridinations of ylides. The first remarkable reports were made by Ishikava’s group on reactions of guanidinium ylides with aryl aldehydes as a practical route for obtaining of inactivated 3-arylated aziridine-2-carboxylates **1a1** [72,73,74,75,76]. The initial study [72] for the first time reported the formation of guanidinium ylides **21b** from guanidinium salts **21a** in the presence of base (NaH or tetramethylguanidine) and their reactions with aryl aldehydes to form *trans* aziridines **1a1** (Scheme 18). In the subsequent publication [73], the potential reaction mechanism and role of the *p*-substituents in aldehyde aryl ring were explored. The authors concluded that in the case of EDG *p*-substituted benzaldehydes S_N_i-like mechanism and in case of EWG substituents S_N_2-type mechanism took place. Not only *trans* aziridines **1a1** are available with this method. Procedure of epimerization in β- (C3) position was described [74] using indium chloride catalyst. Aziridinomitosene skeleton synthesis [74], formal synthesis of (-)-podophyllotoxin [75] and synthesis of cyclic dipeptide (-)-benzolactam-V8 [76] has been demonstrated.

Finally, reaction conditions were optimized, the method was expanded to broad spectrum of aryl aldehydes (45 examples) and series of modified guanidine salts were examined to reach a general method for highly substituted 3-arylaziridine-2-carboxylate **1a1** synthesis [77].

**Scheme 19:**
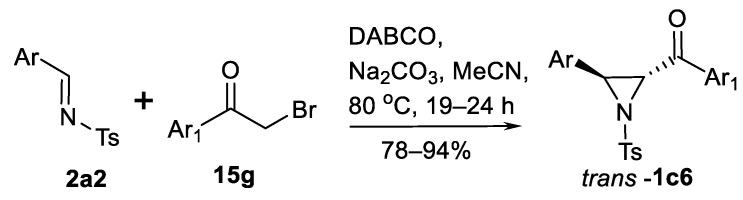
DABCO-promoted aziridination [78].

Other ylides can work in a similar way. Thus, aziridinations via ammonium [78,79,80] and sulfonium [81,82,83,84] ylides are known. A simple protocol for the reaction of phenacyl bromides **15g** with imines **2a2** promoted by tertiary amine (DABCO) via in situ generated ylide (Scheme 19) has been reported [78].

This one-pot aziridination process include quaternization of DABCO, then in situ formation of ammonium ylide in the presence of base and aziridination of imine **2a2** to obtain 3-arylated *trans*-aziridinyl ketones *trans*-**1c6** in good yields (nine examples) and *trans*-selectivity. Enantioselective aziridination using chiral DABCO analogue also was demonstrated.

**Scheme 20:**
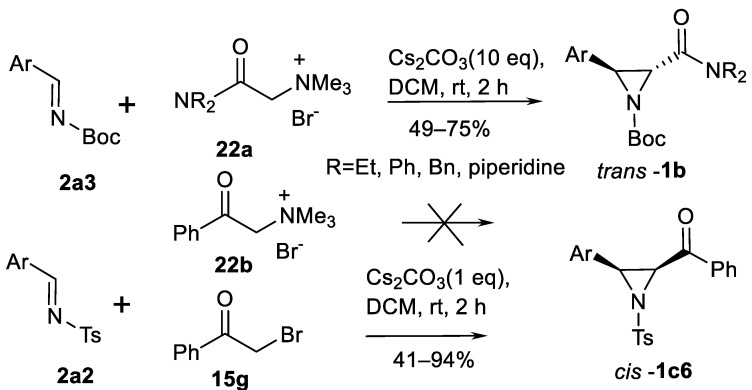
Ammonium salt-promoted aziridination via ylides [79].

Simple trimethylammonium salts work similarly via amide-stabilized ammonium ylides (forming from salts **22a**; Scheme 20) which react with aromatic aldimines **2a3** to form 3-arylated *trans*-aziridine carboxamides *trans*-**1b** [79]. Moderate to good yields and *trans*-selectivity has been demonstrated in eight examples. Remarkable feature is that ammonium salts **22b** do not react with aldimines **2a2** in similar way. However, the classic *aza*-Darzen process—reaction of imines **2a2** with phenacyl bromide **15g**—gives *cis–*aziridinyl ketones *cis*-**1c6** in high yields.

**Scheme 21:**
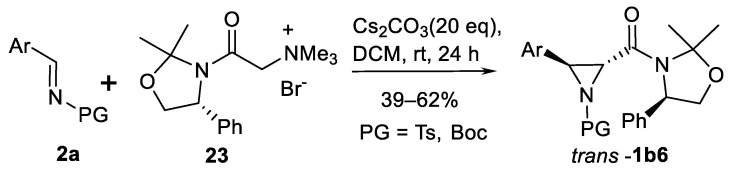
Aziridination with chiral stabilized trimethylammonium salt [80].

Asymmetric aziridination using stabilized trimethylammonium salts **23** has been demonstrated [80] in six examples (Scheme 21). Aziridine carboxamides *trans*-**1b6** were obtained in good yields.

**Scheme 22:**
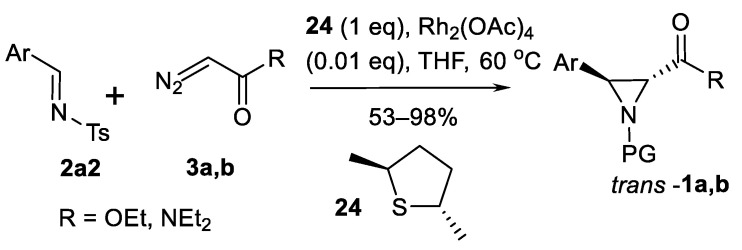
Aggarwal’s aziridination [81,82].

Sulfonium ylide-mediated catalytic asymmetric aziridination of imines were mentioned by Aggarwal’s group [81,82]. Chiral sulfides, for example, **24** and catalytic amounts of metal salts, promote aziridination of imines with diazo compounds via sulphur ylide intermediates (Scheme 22). Five examples of synthesis of aziridines *trans*-**1a, b** were demonstrated reaching good yields (53–98%) and moderate enantioselectivity of 30–58% ee [81].

**Scheme 23:**
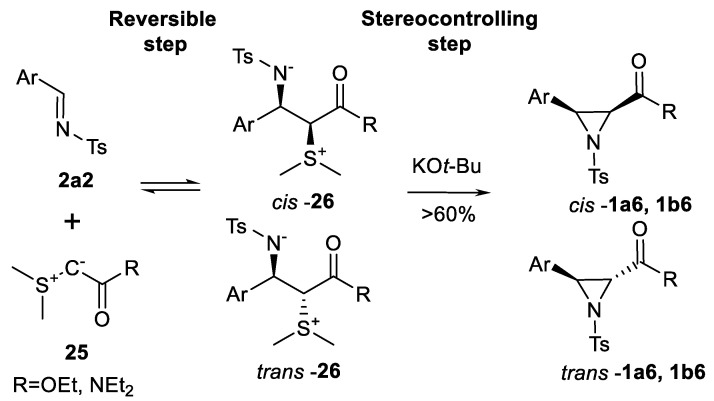
Sulfur ylide aziridination [82].

Ester and amide-stabilized sulfur ylides generated form sulfonium salts were explored [82] as sources of aziridines (Scheme 23). It was established that ester and amide-stabilized sulfur ylides **25** react with activated aryl aldimines **2a2** reversibly to form betaines **26** and the stereocontrolling step is represented by the base-controlled aziridine ring closure leading to aziridines **1a6**, **1b6**.

**Scheme 24:**
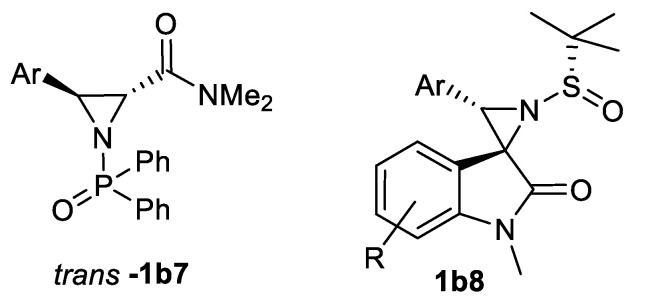
Sulfur ylide aziridination products [83,84].

A series of 3-arylated N-diphenylphospinoyl aziridine-2 carboxamides *trans*-**1b7** are synthesized in similar route from amide sulfonium salts via type **25** stabilized ylides [83] as well as series of chiral 3-aryl spiro-aziridine oxindoles **1b8** (11 examples, 60–76%, dr > 99:1) from corresponding imines using ylides generated from sulfonium salts in presence of NaH (Scheme 24) [84]. Therefore, sulfonium ylides as well as the above mentioned guanidinium and ammonium ylides are useful tools in target aziridine synthesis.

**Scheme 25:**
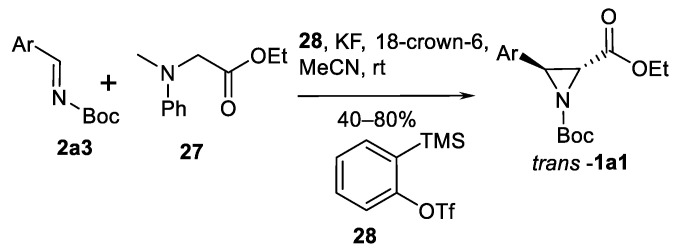
Benzyne-promoted Darzen-type aziridination [85].

An interesting variation of aziridine synthesis from imines is the benzyne-promoted Darzen-type reaction of tertiary amine **27** as shown in the Scheme 25 [85]. This process was performed in mild conditions (no strong bases, room temperature) in the presence of 2-(trimethylsilyl)phenyl triflate **28**, KF and a crown ether. Five different 3-arylated aziridine-2-carboxylates *trans*-**1a1** were obtained in moderate to good yields (40–80%) and good *trans*-selectivity > 98:2 dr.

## 3. Aziridination of Olefins (Path B)

This pathway of aziridination includes:

- Evans aziridination with arylsulphonyliminophenyliodinanes;
- Oxidative aziridination with N-aminophtalimide and its analogues;
- Active hydroxylamines as aminating agents.

### 3.1. Evans Aziridination

**Scheme 26:**
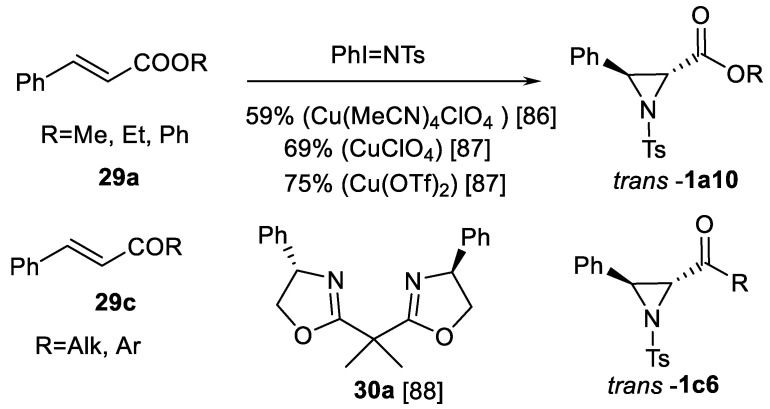
Evans aziridination [86,87,88,89].

The reaction of olefins with active nitrene species generated from various nitrene precursors represents another well-known and reliably explored method to synthesize aziridine structures **1**. The first approach, notable for 3-aryl aziridine-2-carboxylic acid derivative synthesis, is the classical Evans aziridination using PhI=NTs as nitrene precursor, cinnamate type substrates **29** as olefins in the presence of Cu salts as catalysts (Scheme 26) [86,87].

Other nitrene precursors (*p*-NO_2_ and *p*-OMe benzenesulphonylimino-phenyliodinanes instead of PhI=NTs) may increase yields of **29a** type cinnamate aziridinations products **1a10** to 97% in 87% ee using chiral ligand **30a** (BOX) [88]. Aziridination using iodosylbenzene in combination with sulfonylamides instead of iminoiodinanes has been reported, but the yields of **1a** type esters were only moderately high reaching 40–53% [89].

**Scheme 27:**
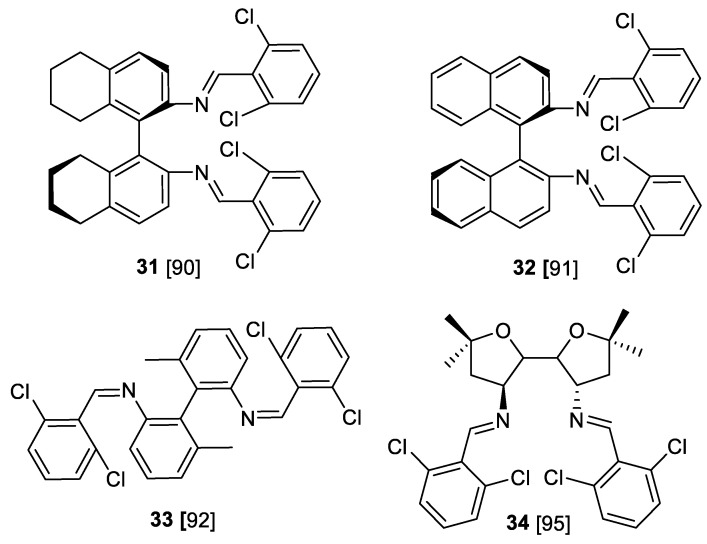
Chiral ligands for Evans aziridination [90,91,92,95].

The further investigations in Evans aziridination included various design of chiral ligands (Scheme 27). Thus, a series of cinnamates were aziridinated using copper-catalyzed asymmetric aziridination with PhI=NTs and chiral salen type ligand **31** (nine examples, yield 60–90%, 61–93% ee) [90] as well as binaphthyldiimine ligand **32** (11 examples, yield 47–92%, 11–97% ee) [91]. A series of cinnamates and chalcones has been successfully aziridinated by PhI=NTs/[Cu(MeCN)_4_]PF_6_ system in the presence of the same ligands **32** (*R* and *S* BINIM-DC) (15 examples, yields 41–87%, 36–97% ee) [92].

Similarly, biaryl Shiff base **33** was used as a ligand (nine examples, yield 32–77%, 89–98% ee) [93]. Aziridine esters *trans*-**1a** have been obtained, and structures of copper-ligand **32** complexes [93] and reaction mechanisms [94] have been explored. The improved diimine ligand **34** was used in asymmetric aziridination of cinnamates under **34**/Cu(1) catalysis with PhI=NTs as nitrene source (yield 63–99%, >80% ee) or in a one-pot procedure with iodobenzene diacetate-sulfonamide system [95].

**Scheme 28:**
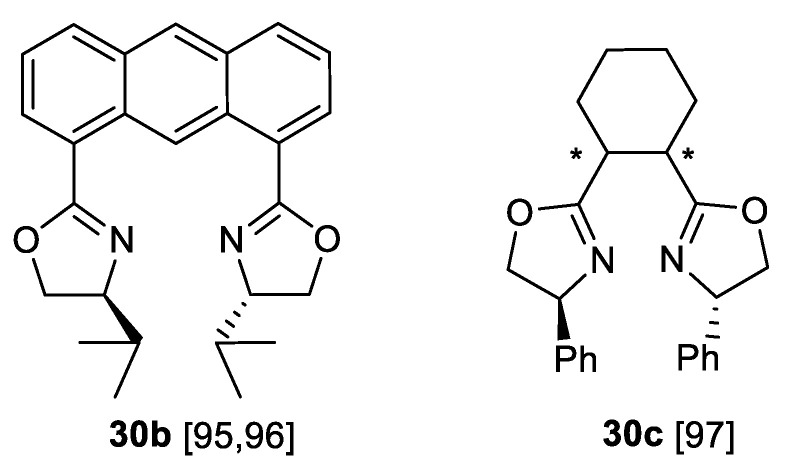
AnBOX and cHBOX ligands [95,96,97].

The next well-explored group of chiral ligands for Evans-type aziridination of olefins that are used in synthesis of 3-arylaziridine-2-carboxylic acid derivatives **1a** are bidentate bis-oxazolinyl type compounds—the analogues of BOX ligand **30a** mentioned above [88]. The further development of such ligands are 1.8-bisoxazolinylanthracene (AnBOX) type ligands **30b** [96,97] and cyclohexane-linked bis-oxazolines **30c** (cHBOX) shown in the Scheme 28 [98]. The AnBOX ligand **30b** was successfully used in asymmetric aziridination of chalcone type substrates **29c** with PhI=NTs in CuOTf catalysis to obtain aziridinyl ketones *trans*-**1c** (nine examples, yields 51–91%, 68–99% ee) [96].

In a subsequent, more detailed study [97] the substrate scope was expanded (22 chalcone and a single cinnamate substrate example, yield 35–92%, 27–99% ee) and the limitations explored. Higher enantioselectivity in the chalcone aziridination has been reached using (*S*)-cHBOX **30c** (11 examples, yield 51–73%, >80% ee) [98].

The comparison of BOX **30a**, AnBOX **30b** and cHBOX **30c** ligands in Evans-type chalcone substrate **29c** aziridination and the exploration of π-stacking between chalcone and ligand aromatic systems were performed [99]. The results showed that π–interaction between chalcone substrates **29c** and the AnBOX ligand’s **30b** anthracene backbone was important in order to improve the enantioselectivity of aziridination. 

The further improvements of Evans chalcone **29c** and cinnamate **29a** aziridination included use of Cu(2) and poly/perfluorinated alkoxyaluminate type anion complexes [100], alumina-supported [101] and immobilized magnetic Cu containing nanoparticles [102]. Use of gold instead of copper catalyst has also been reported [103].

In conclusion, Evans aziridination is a practical method for the synthesis of *trans*-3-arylated aziridine-2-carboxylates **1a** and aziridin-2-ylketones **1c** from corresponding cinnamates **29a** and chalcones **29c**, respectively.

### 3.2. Oxidative Aziridination

**Scheme 29:**
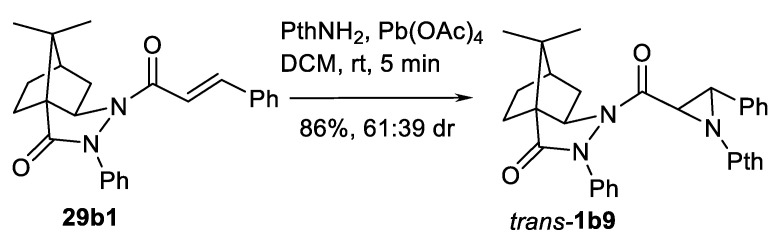
Oxidative aziridination of a chiral substrate [104].

Another important method for asymmetric aziridination of alkenes is the reaction with N-aminophthalimide in the oxidative conditions (Pb(OAc)_4_ or another oxidant) as a nitrene source. The asymmetric induction can be conducted with chiral moiety in the substrate and with chiral ligand. Thus, use of chiral auxiliary can be illustrated by aziridination of chiral camphor N-enoylpirazolidinone **29b1** to obtain aziridine-2-hydrazide *trans*-**1b9** (Scheme 29) in good yield [104]. Another chiral camphor-based auxiliary-directed aziridination of aziridine esters was demonstrated [105] but this study was focused on only a single example of cinnamate **29a**.

**Scheme 30:**
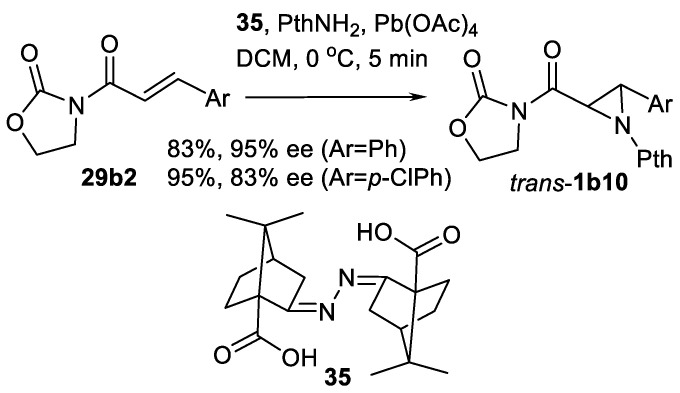
Oxidative aziridination using a chiral ligand [106].

Chiral ligand-mediated variation of this aziridination was studied by the same authors [106]. Reaction of N-enoyl oxazolidinones **29b2** in similar conditions and in presence of ligand **35** lead to aziridine-carboxamides *trans*-**1b10** in good yields and >80% ee (Scheme 30).

Similar Pb(OAc)_4_-mediated aziridination with PthNH_2_ as nitrogen source using chalcone and cinnamate type substrates was successfully employed to obtain aziridine intermediates for oxazole synthesis [107,108], spiro-fused N-phthalimidoaziridines [109] and 3(2-allylphenyl)aziridine-2-carboxylates [110] to investigate their thermal transformations.

**Scheme 31:**
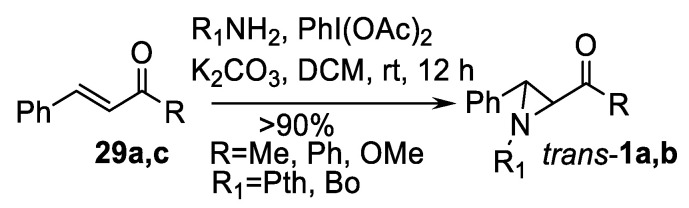
Iodobenzene diacetate-promoted oxidative aziridination [111].

Other oxidant systems used in this type of alkene aziridination include iodobenzene diacetate [111] and aril iodide-*m*-CPBA system [112]. Iodobenzene diacetate works well as oxidant in the aziridination of chalcones **29c** and cinnamate **29a** with N-aminophtalimide (PthNH_2_) or 3-amine-3H-benzoxazol-2-one (BoNH_2_) as nitrogen sources. Yields of aziridines *trans*-**1a**,**b** (Scheme 31) are excellent [111].

**Scheme 32:**
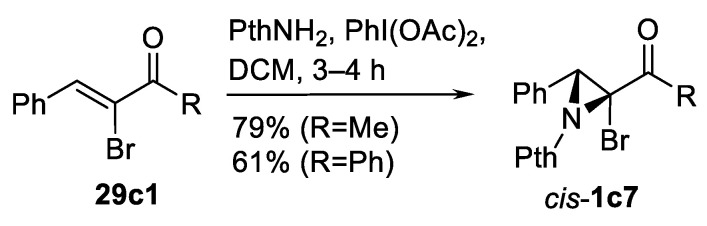
Yudin’s modification of oxidative aziridination [113].

The same transformation using in situ generated oxidant made from *m*-CPBA and 4-methoxyphenyl iodide was demonstrated [112] and compared with PhI(OAc)_2_ oxidant in series of alkene substrates including chalcone **29c**. The yields were comparable. Interesting utilization of PthNH_2_-PhI(OAc)_2_ aziridination was demonstrated by Yudin’s group [113]; this system allowed them to obtain 3-phenyl-2-bromoaziridinylketones *cis*-**1c7** from corresponding α-bromoketones **29c1** in good yields (Scheme 32).

**Scheme 33:**
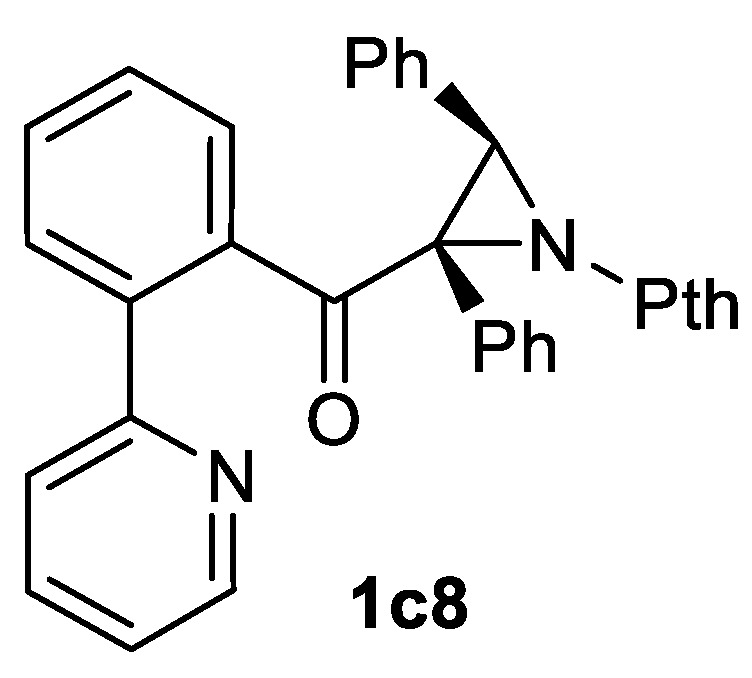
An example of a highly functionalized product of oxidative aziridination [116].

Aziridination of highly functionalized chalcone and cinnamate type substrates with a PthNH_2_ nitrogen source and a PhI(OAc)_2_ oxidant was utilized for functionalized 3-benzazepine skeleton construction [114] and for obtaining of fluorinated aziridines including 3-C_6_F_5_ substituted aziridine-2-carboxylate **1a** [115]. Example of highly functionalized biaryl aziridinyl ketone **1c8** (Scheme 33) synthesis from corresponding alkene via the PthNH_2_/PhI(OAc)_2_ system has been demonstrated [116].

**Scheme 34:**
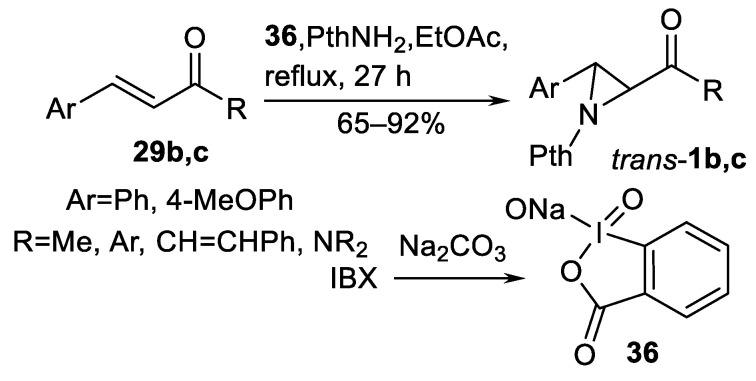
Sodium 2-iodoxybenzoate-promoted oxidative aziridination [117].

An interesting and perspective oxidant in oxidative aziridination is sodium 2-iodoxybenzoate **36** generated from *o*-iodoxybenzoic acid (IBX) and Na_2_CO_3_ (Scheme 34) [117]. Various type **29** substrates including unsaturated ketones **29c** and amides **29b** has been successfully aziridinated to obtain *trans*-**1b, c** type products in 65–92% yields (five examples).

**Scheme 35:**
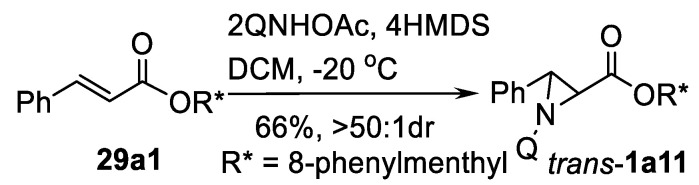
QNOAc-promoted aziridination [118].

Another nitrogen source for substrate-controlled diastereoselective aziridination is 3-acetoxyaminoquinazolone (QNHOAc) in the presence of hexamethyldisilazane (HMDS) as shown in the Scheme 35 [118]. 

**Scheme 36:**
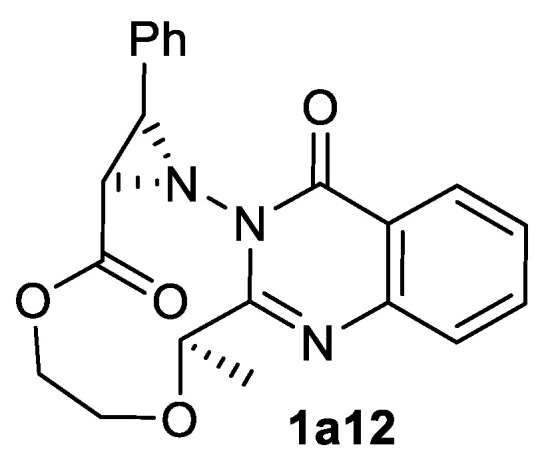
The product of intramolecular aziridination [119].

Aziridine ester *trans*-**1a11** was obtained in good yield and diastereomeric excess from cinnamate **29a1**. Intramolecular variation of this process also has been reported in the synthesis of the complex aziridine ester **1a12** depicted in the Scheme 36 [119].

**Scheme 37:**
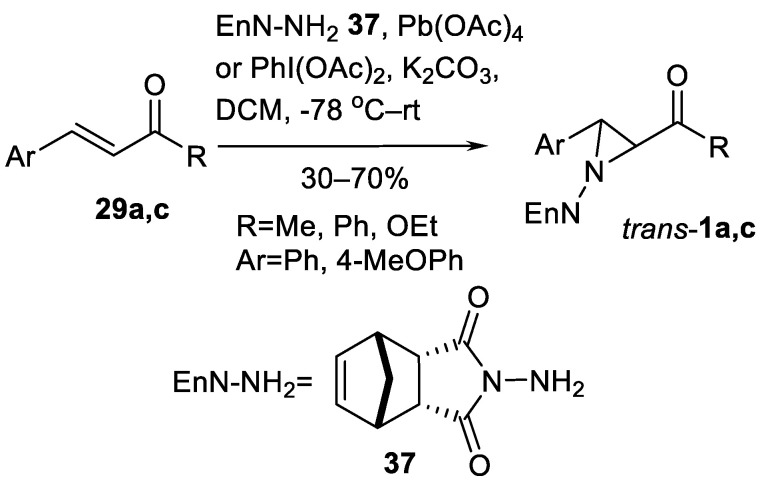
EnH-NH_2_-promoted oxidative aziridination [120].

An interesting nitrogen source (N-amino-*endo*-bicyclo[2.2.1]hept-5-ene-2.3-dicarboxylic acid diimide **37** (EnH-NH_2_)) was investigated (Scheme 37) in four examples, leading to the yields of 30–70% [120].

**Scheme 38:**
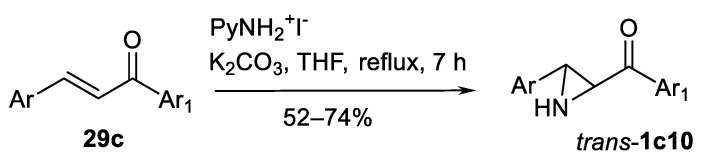
1-Aminopyridinium iodide as the nitrogen source in aziridination [121].

1-aminopyridinium iodide (Scheme 38) represented another original nitrogen source [121].

This NH-transferring agent allows to obtain NH aziridinylketones *trans*-**1c10** from chalcones **29c** in 52–74% yield (11 examples) in a stereoselective one-pot process.

**Scheme 39:**
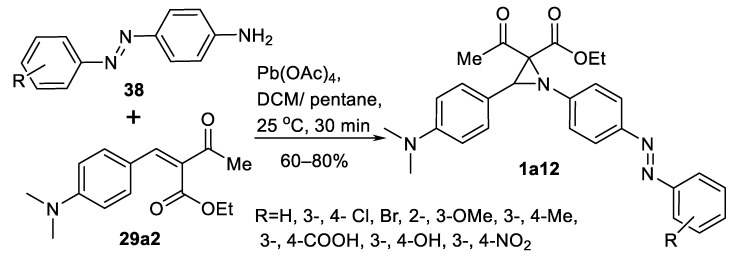
Anilines as the nitrogen source in oxidative aziridination [122].

An example of substituted anilines **38** as nitrogen sources in oxidative aziridination of benzilidene dicarbonyl substrate **29a2** to obtain potential antibacterial aziridines **1a12** (Scheme 39) has been reported [122]. Aziridination was carried out at room temperature, and the yields of aziridines **1a12** were good (60–70%) in 15 examples.

Lead or hypervalent iodine oxidants in oxidative aziridination with PthNH_2_ as a nitrogen source can be replaced with direct electrochemical oxidation via +1.80 V potential [123]. Methyl cinnamate **29a** and chalcone **29c** have been aziridinated in yields of type *trans*-**1a**,**c** aziridines 86% and 83%, respectively.

**Scheme 40:**
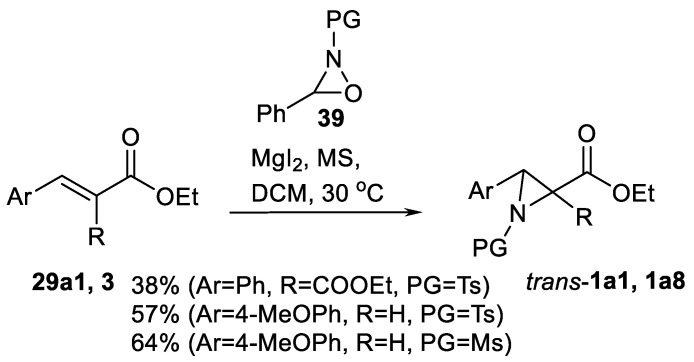
Oxaziridine-promoted oxidative aziridination [124].

Oxaziridine **39** can be used simultaneously as oxidant and nitrogen source for oxidative nitrogen transfer alkene aziridinations in synthesis of 3-arylaziridine-2-carboxylates **1a1**, and diesters **1a8** as shown in the Scheme 40 [124]. The reaction was carried out under mild conditions in presence of MgI_2_.

**Scheme 41:**
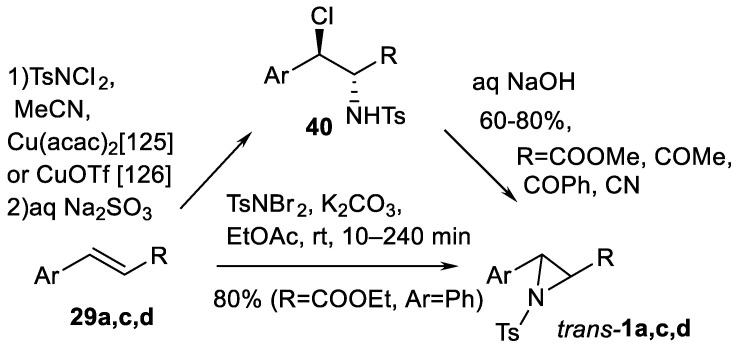
N,N-dihalogene-*p*-toluenesulfonamides as the nitrogen source in aziridination [125,126,127].

N, N-dihalogene-*p*-toluenesulfonamides are reported as nitrogen sources for both catalytic [125,126] and non-catalytic [127] aziridinations of cinnamates, cinnamyl amides and chalcone type substrates **29a**,**c**,**d** (Scheme 41). Interestingly, aziridination with TsNCl_2_ was demonstrated as a two-step addition-cyclization process [125,126] via isolable intermediate **40**, but the reaction with TsNBr_2_ was reported in a one-step nitrene transfer process [127]. The specific use of similar aziridinating agent PhSO_2_NBr_2_ under Lu(OTf)_3_/chiral ligand catalysis for complex tricyclic aziridine synthesis was noted [128].

**Scheme 42:**
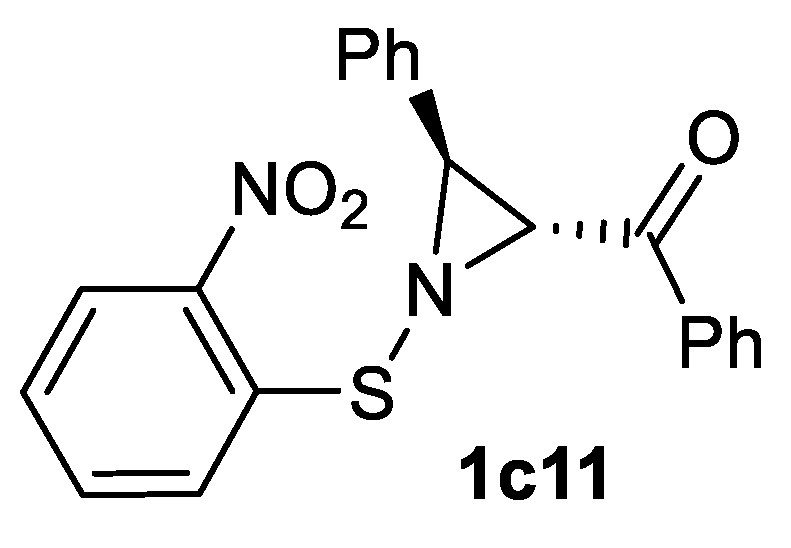
An example of N-sulfenylaziridine product [129].

The other nitrene sources are sulfenyl nitrenes generated from N-sulfenylsulfodiimides [129] and bromamine T [130]. Sulfenyl nitrenes allow to obtain N-sulfenylaziridines, a single example of N-sulfenylaziridine **1c11** has been demonstrated (60% yield from chalcone [129]) (Scheme 42).

Bromamine T is moderately effective as nitrene source for cinnamate substrates **29a** (Scheme 41) under ultrasound conditions in the presence of CuCl_2_ forming aziridine-2-carboxlates *trans*-**1a** (30–34% yield) and under microwave in the presence of CuBr_2_ forming esters *cis*+*trans*-**1a** (Scheme 41) at 36–38% yield [130].

### 3.3. Hydroxylamines as Nitrogen Sources

**Scheme 43:**
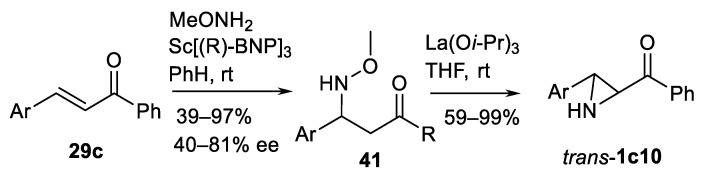
Michael-type addition-based aziridination [131].

Classically, aziridines can be synthesized from conjugated α,β-unsaturated substrates by the Michael-type addition followed by aziridine ring closure, usually under basic conditions. Thus, chalcone type substrates **29c** (Scheme 43) undergo Michael addition of O-methylhydroxylamine under scandium (*R*)-1.1-binaphtyl-2.2′-diyl phosphate (BNP) complex catalysis and then perform cyclization of intermediate **41** into aziridines *trans*-**1c** under lanthanum (3) isopropoxide catalysis as shown in the Scheme 43 [131]. Remarkably, this is a route to 3-arylated NH-aziridin-2-ylketones *trans*-**1c10**. In case of racemic intermediate **41**, a kinetic resolution using La(O*i*-Pr)_3_-mediated cyclization into aziridine *trans*-**1c10** in the presence of (*R*) or (*S*)-BINOL is possible.

**Scheme 44:**
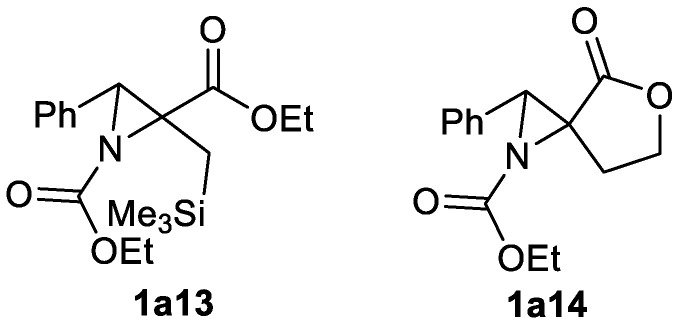
Carbamate reagent-based aziridination products [132,133].

Highly functionalized aziridine ester **1a13** (yield 58%) [132] and lactone **1a14** (yield 60%) [133] can be produced from corresponding α,β–unsaturated **29a** type carboxylic ester and lactone, respectively, in reaction with carbamate reagent NsONHCOOEt in the presence of CaO (Scheme 44).

**Scheme 45:**
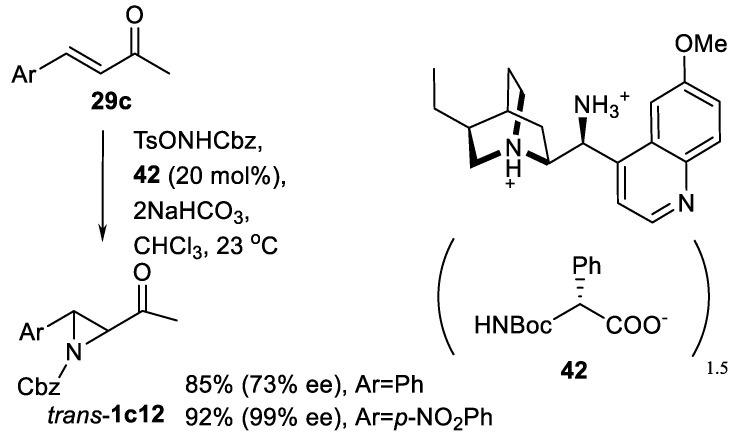
Asymmetric organocatalytic aziridination of enones with carbamate reagent [134].

Similar asymmetric organocatalytic aziridination of enones with carbamate reagent TsONHCbz in the presence of catalyst salt **42** (Scheme 45) has been reported [134]. Aziridinyl ketones *trans*-**1c12** were obtained in good yields and ee.

**Scheme 46:**
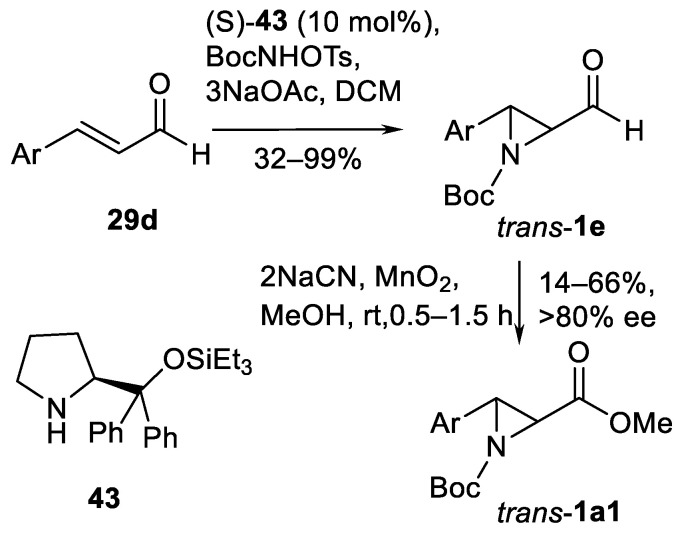
Enantioselective organocatalytic aziridination of α,β–unsaturated aldehydes [135].

In the same way, employing TsONHBoc reagent and chiral diphenylprolinol triethylsilyl ether **43** organocatalyst, enantioselective organocatalytic aziridination of α,β-unsaturated aldehydes **29d** (Scheme 46) can be performed [135].

**Scheme 47:**
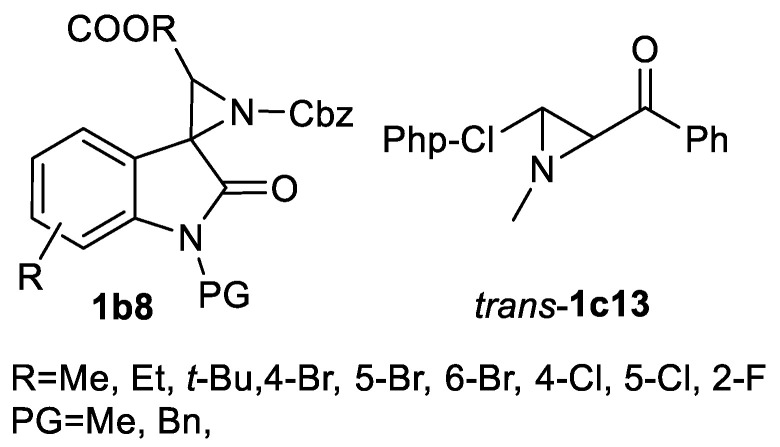
Hydroxylamine reagent-mediated aziridination products [137,138].

Synthesis of various 3-arylated aziridine aldehydes *trans*-**1e** and corresponding esters *trans*-**1a1** (Scheme 46) has been demonstrated in 18 examples showing good yields and enantioselectivity (>80% ee). Aryl substituents include various substituted phenyls, fluorinated phenyls and 3-piridyls. Enantioselective synthesis of (*R*)-sumanirole employing this aldehyde **29d** aziridination method was reported [136]. The syntheses of spiroaziridine oxindoles **1b8** (13 examples, 74–98% yields) are performed using BocONHCbz hydroxylamine in the presence of tetramethylguanidine [137]. An isolated example of inactivated aziridin-2-ylketone *trans*-**1c13** (Scheme 47) (60% yield) in TsONHMe-mediated aziridination under Rh_2_(esp)_2_ catalysis (Scheme 47) was demonstrated [138].

**Scheme 48:**
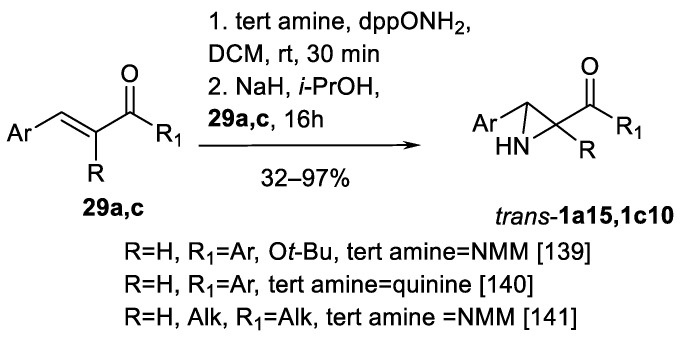
Armstrong’s aziridination [139,140,141].

Finally, Armstrong’s aziridination must be noted as a remarkable advance in the direct access to 3-arylated N-unsubstituted aziridin-2-yl ketones *trans*-**1c10** (Scheme 48) and carboxylates *trans*-**1a15.** Armstrong’s aziridination implies the use of N,N-ylides generated from N-methylmorpholine, or other tertiary amines in the presence of Ph_2_P(O)ONH_2_ (dppONH_2_) as NH transfer agent (Scheme 48) [139,140,141].

In the initial work [139], chalcones and cinnamates **29a, c** were successfully aziridinated to obtain *trans*-aziridine-2-ketones **1c10** and carboxylates *trans*-1a15 (Scheme 48) using N-methylmorpholine. The yields were 32–97%; higher yields were obtained by chalcones **29c**. Enantioselective variation of this process using quinine as tertiary amine (yields 35–68% and 37–56% ee, eight examples) [140] and aziridination of highly functionalized alkyl arylidene ketones **29c** (Scheme 48) (yields 51–81%) has been reported [141].

## 4. Conclusions

Aziridination has a great synthetic potential in synthesis of 3-arylated aziridine-2-carboxylates, carboxamides and 2-aziridinylketones. The most important, well-explored, and practical in terms of imine C=N bond aziridination are Wulff’s catalytic AZ reaction employing diazo compounds as carbene sources and various catalytic systems.

This method demonstrates a high stereoselectivity in a broad series of examples and allow obtaining both *cis* and *trans* aziridine products including nitriles and aldehydes selectively. In C=C bond aziridination, the main approaches include Evans olefin aziridination using PhI=NTs type nitrene sources under Cu catalysis, as well as oxidative aziridination variations.

Notably, Evans aziridination is suitable for aziridination of chalcone and cinnamate type substrates and oxidative methods allows to obtain also 3-arylaziridine-2-carboxamides. Remarkable are several methods which allow to directly access NH aziridines, such as Armstrong’s aziridination.
